# Critically Ill Setting / Productivity Grid (CISPG): Proposal For Distribution of Productive Time of Intensivists in a Critical Care Medicine Department (CCMD) Considering 'Outreach’

**DOI:** 10.1186/2197-425X-3-S1-A481

**Published:** 2015-10-01

**Authors:** J Ruiz Moreno, JM Nicolás Arfelis, V Gómez Tello, MJ Esteve Paños, R Corcuera Romero de la Devesa, E González Marín, P Castro Rebollo, P Merino de Cos, N Franco Garrobo, F Baigorri González

**Affiliations:** Quirón Salud Hospital Universitario Sagrat Cor, Critical Care Department, Barcelona, Spain; Hospital Clinic de Barcelona, Critical Care Department, Barcelona, Spain; Moncloa Hospital Universitario, Critical Care Department, Madrid, Spain; Hospital Can Misses, Critical Care Department, Ibiza, Spain; Hospital Universitario Móstoles, Critical Care Department, Móstoles, Spain

## Introduction

The distribution of intensivists productive time is a classical controversial subject. However it is true that such a professional can practice their profession both within and outside (outreach) the ICU. It is also true also that intensivists serve patients, teaching and research, as well as consider the clinical management (CM) and the risk management (RM).

## Objectives

To propose a CISPG to distribute the productive time of intensivists both in the ICU and outreach.

## Methods

Setting - CCMD A) level III, 14 beds; and CCMD B) level IIb, 10 beds.

Definition of ‘productivity’: 1. Assistance (1.1. ICU assistance and 1.2. SDU assistance. 2. Teaching. 3. Research. 4. Risk management (RM). 5. Clinical management (CM).

CCMDs features:

CCMD A: 8 ICU beds and 6 SDU beds. ICU assistance, outreach assistance, teaching, experimental and epidemiological research, RM, and CM.

CCMD B: 6 ICU beds and 4 SDU beds. ICU assistance, outreach assistance, teaching, epidemiological research, RM, and CM.

CISPG:

Abscissas (productivity between 08:00 and 17:00): ICU assistance, outreach, teaching, research, RM, and CM.

Ordinates (setting): ICU, SDU, hospital ward (pre and postcritically iIl), emergency room (precritically ill and CIP)

3 medical professional job categories: senior, chief clinical (CC) and head of CCMD (HD).

Physician on call are not considered.

Annual working time per physician: 1750 hours

Times adjusted to each specific productivity (sum total % = 100).

It is established 1 HD, 1 CC for attending CCMDs a and B, and respectively, 4 and 3 seniors.

## Results

## Conclusions

The MAPEC facilitates the distribution of specific work time for intensivists attending both the setting and structured productivity.

**Table 1:** CISPG.

Setting	Hours	Assistance %	Teaching %	Research %	CM %	RM %
ICU Assistance Teaching Research CM RM	ICU - 8050 Assist - 3675 Teach - 875 Resear- 875 CM - 1225 RM - 1400	Senior 1 60 Senior 2 60 Senior 3 60 Cl. chief 20 Head Dp 20	Senior 1 10 Senior 2 10 Senior 3 10 Cl. chief 10 Head Dp 10	Senior 1 10 Senior 2 10 Senior 3 10 Cl. chief 10 Head Dp 10	Senior 1 5 Senior 2 5 Senior 3 5 Cl. chief 15 Head Dp 40	Senior 1 15 Senior 2 15 Senior 3 15 Cl. chief 20 Head Dp 15
SDU Assistance Teaching Research CM RM	SDU -1487 Assist -787 Teach - 175 Resear- 175 CM - 87 RM - 262	Senior 4 30 Cl. chief 10 Head Dp 10	Senior 4 10	Senior 4 10	Senior 4 5	Senior 4 15
Hosp. ward	Hosp. ward 525	Senior 4 15 Cl. chief 10 Head Dp 5				
Emergency	Emergency 437	Senior 4 15 Cl. chief 10 Head Dp 5				
Global hours	10500	5425	1050	1050	1312	1662
